# Supplementary material for: Boosting GPT models for genomics analysis: generating trusted genetic variant annotations and interpretations through RAG and Fine-tuning
Source: Bioinform Adv. 2025 Feb 5;5(1):vbaf019. doi: 10.1093/bioadv/vbaf019 (PMC11842050; doi:10.1093/bioadv/vbaf019)
Supplement: vbaf019_Supplementary_Data [file vbaf019_supplementary_data.docx]

**Supplementary Table 1**. Command-line scripts and sample data used for this analysis

| Description | Code / Data |
| --- | --- |
| Variant functional effect prediction | java -Xmx8g -jar snpEff.jar -v -stats clinvar.html GRCh38.mane.1.2.ensembl clinvar_20240603.noinfo.vcf > clinvar_20240603.noinfo.snpEff.vcf |
| Fine-tuning prompt generation | bcftools query clinvar_20240603.vcf.gz -f "%CHROM\t%POS\t%REF\t%ALT\t%INFO/RS\t%ID\t%INFO/ALLELEID\t%INFO/CLNHGVS\t%INFO/MC\t%INFO/CLNSIG\t%INFO/CLNREVSTAT\t%INFO/GENEINFO\t%INFO/CLNDN" \| awk '{split($9, a, "\|");split($12, b, ":"); print "{\"messages\": [{\"role\": \"system\", \"content\": \"You are an expert genomics annotator.\"}, {\"role\": \"user\", \"content\": \"Name:chr"$1"_"$2"\|Task: Provide full annotation information for the given Name including Gene(s),Protein change,Condition(s).\"}, {\"role\": \"assistant\", \"content\": \"GRCh38 Chromosome: chr"$1"\|GRCh38 Location: "$2"\|REF: "$3"\|ALT: "$4"\|dbSNP ID: rs"$5"\|VariationID: "$6"\|AlleleID(s): "$7"\|Canonical SPDI: "$8"\|Molecular consequence: "a[2]"\|Germline classification: "$10"\|Germline review status: "$11"\|Gene: "b[1]"\|Condition(s): "$13"\"}]}"}' \| sed "s/rs./nan/g"\|less > clinvar_20240603.jsonl |
| Fine-tuning prompt example | {  "messages":  [  {"role": "system",  "content": "You are an expert genomics annotator."},  {"role": "user",  "content": "Name:chr1_69134\|Task: Provide full annotation information for the given Name including Gene(s),Protein change,Condition(s)."},  {"role": "assistant",  "content": "GRCh38 Chromosome: chr1\|GRCh38 Location: 69134\|REF: A\|ALT: G\|dbSNP ID: nan\|VariationID: 2205837\|AlleleID(s): 2193183\|Canonical SPDI: NC_000001.11:g.69134A>G\|Molecular consequence: missense_variant\|Germline classification: Likely_benign\|Germline review status: criteria_provided,_single_submitter\|Gene: OR4F5\|Condition(s): not_specified"}  ]  } |
| RAG data preparation | bcftools query clinvar_20240603.vcf.gz -f "%CHROM\t%POS\t%REF\t%ALT\t%INFO/RS\t%ID\t%INFO/ALLELEID\t%INFO/CLNHGVS\t%INFO/MC\t%INFO/CLNSIG\t%INFO/CLNREVSTAT\t%INFO/GENEINFO\t%INFO/CLNDN" \| awk '{split($12, b, ":"); gsub(/,/, ";", $13); print "chr"$1":"$2",rs"$5","b[1]","$13",\"GRCh38_chr:chr"$1"\tGRCh38_pos:"$2"\treference_allele:"$3"\talternative_allele:"$4"\tdbSNP_ID:rs"$5"\tVariation_ID:"$6"\tAllele_ID:"$7"\tcanonical_SPDI:"$8"\tmolecular_consequence:"$9"\tgermline_review:"$10"\tgermline_status:"$11"\tGene:"b[1]"\tCondition:"$13"\tsource:clinvar\tclinvar_URL:https://www.ncbi.nlm.nih.gov/clinvar/variation/"$6"/\""}' \| sed "s/rs\./na/g" \| sed "s/\"See_Cases\"/See_Cases/g" > clinvar_20240603.5col.csv |
| Sample input data for RAG | chr1:69134,na,OR4F5,not_specified,"GRCh38_chr:chr1 GRCh38_pos:69134 reference_allele:A alternative_allele:G dbSNP_ID:na Variation_ID:2205837 Allele_ID:2193183 canonical_SPDI:NC_000001.11:g.69134A>G molecular_consequence:SO:0001583\|missense_variant germline_review:Likely_benign germline_status:criteria_provided,_single_submitter Gene:OR4F5 Condition:not_specified source:clinvar clinvar_URL:https://www.ncbi.nlm.nih.gov/clinvar/variation/2205837/" |

**Supplementary Table 2**. Overview of variant annotation datasets integrated into GPT models through RAG or fine-tuning.

| Dataset/ Tool | No. Variants | Selected Annotation Fields (one example variant) |
| --- | --- | --- |
| ClinVar | 2,897,556 | GRCh38 chr: chr1  GRCh38 pos: 69134  reference allele: A  alternative allele: G  dbSNP ID: na  Variation ID: 2205837  Allele ID: 2193183  canonical SPDI: NC_000001.11:g.69134A>G  molecular consequence: SO:0001583\|missense_variant  germline review: Likely benign  germline status: criteria provided, single submitter  Gene: OR4F5  Condition: not specified  source: ClinVar  ClinVar URL:  https://www.ncbi.nlm.nih.gov/clinvar/variation/2205837/ |
| gnomAD | 183,717,261 | GRCh38 chr:pos: chr1:12948  dbSNP ID: rs1199063229  reference allele: T  alternative allele: C  allele frequence (AF): 0.000367962  AF afr: 0.00523088  AF amr: 0.000373227  AF asj: 0.000291545  AF eas: 0.000439947  AF fin: 0  AF mid: 0.00174825  AF nfe: 0.000132535  AF sas: 8.98796e-05  AF remaining: 0.000959003  source: gnomAD  gnomAD URL: https://gnomad.broadinstitute.org/variant/1-12948-T-C?dataset=gnomad_r4 |
| SnpEff | 2,897,556 | GRCh38 chr:pos: chr1:69134  dbSNP ID: na  reference allele: A  alternative allele: G  allele: G  annotation: missense variant  annotation Impact: MODERATE  gene name: OR4F5  gene ID: ENSG00000186092.7  feature type: transcript  feature ID: ENST00000641515.2  transcript BioType: protein coding  rank: 3/3  HGVS.c: c.107A>G  HGVS.p: p.Glu36Gly  cDNA.pos/cDNA.length: 167/2618  CDS.pos/CDS.length: 107/981  AA.pos/AA.length: 36/326  Distance:  ERRORS/WARNINGS/INFO:  source: snpEff |
| GWAS Catalog | 625,113 | GRCh38 chr:pos: chr3:85356351  dbSNP ID: rs9822731  date added to catalog: 2019-03-18  PubMed ID: 30643258  first author: Karlsson Linner R  journal: Nat Genet  publication link: https://www.ncbi.nlm.nih.gov/pubmed/30643258  disease/ trait: Alcohol consumption (drinks per week)  reported gene: CADM2  mapped gene: CADM2  strongest SNP-risk allele: rs9822731-T  functional effect: intron variant  risk allele frequency: 0.7753  p-value: 4E-15  odds ratio: 0.021035347  source: GWAS Catalog |
| pharmGKB | 41,287 | dbSNP ID: rs75527207  gene: CFTR  level of evidence:1A  drug: ivacaftor  phenotype: Cystic Fibrosis  source: pharmGKB  pharmGKB URL: https://www.pharmgkb.org/clinicalAnnotation/981755803 |


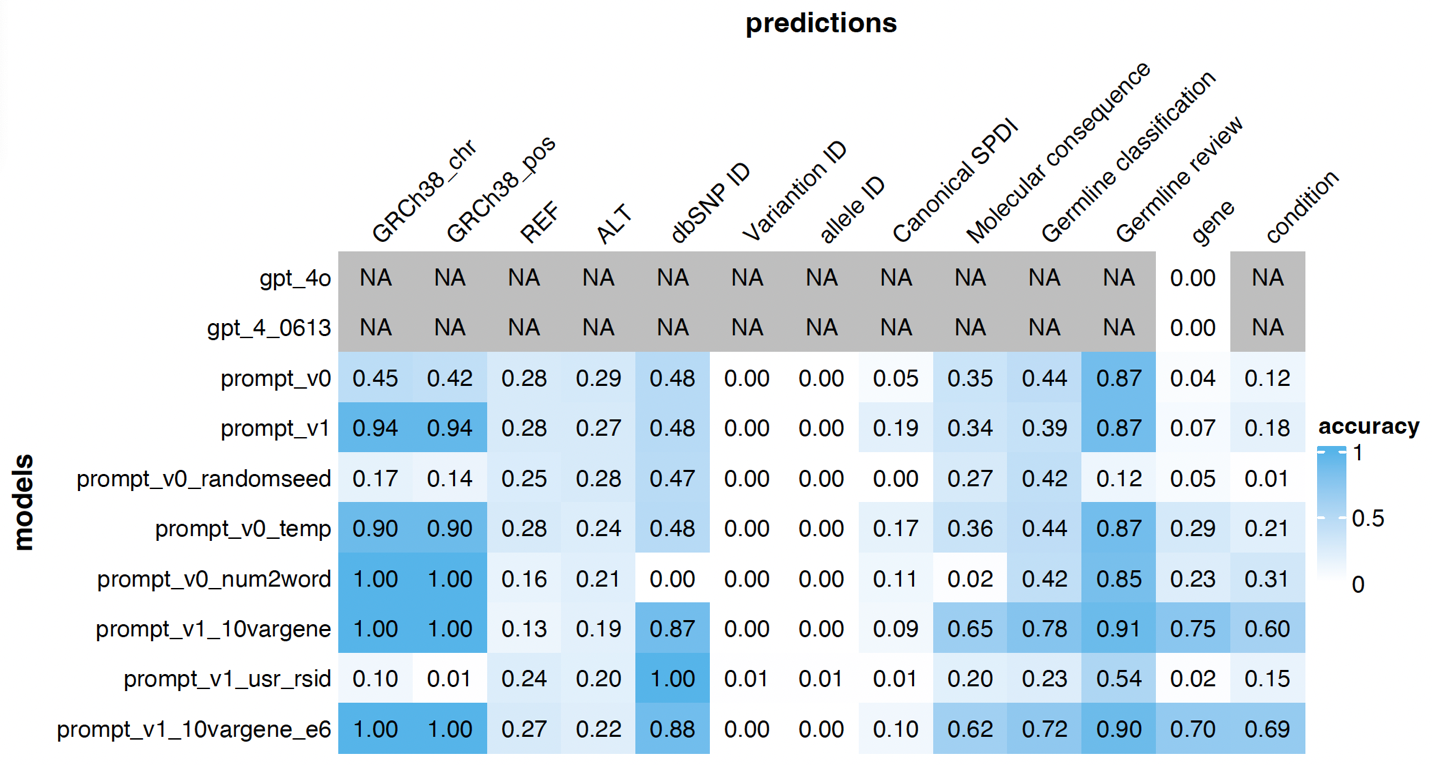


**Supplementary Figure 1**. Fine-tuning GPT-4 model for predicting 13 annotation fields simultaneously. The performance of each model is measured by the accuracy of predicting the exact match of each annotation field. The base GPT-4o and GPT-4 models are unable to correctly predict genes from user provided variants. We fine-tuned the models using diverse input data formats, including: 1) prompt_v0: used the same format as described in the Methods section; 2) prompt_v1: added { } around each annotation field to enhance segmentation; 3) randomseed: tested the model with a different random seed; 4) temp: tests model with higher temperature setting; 5) num2word: encoded numerical IDs (dbSNP ID, Variation ID, and allele ID) as random words to improve tokenization; 6) 10vargene: included 10 variants per gene in the training dataset, 7) usr_rsid: changed user input variants from the chr:pos format to the dbSNP ID; 8) 10vargene_e6: included 10 variants per gene in the training dataset and trained model for an addition 3 epochs, resulting in 6 epochs in total.
